# Integrated metabolic, transcriptomic and chromatin accessibility analyses provide novel insights into the competition for anthocyanins and flavonols biosynthesis during fruit ripening in red apple

**DOI:** 10.3389/fpls.2022.975356

**Published:** 2022-09-23

**Authors:** Chunzhen Cheng, Ziwei Guo, Hua Li, Xiaopeng Mu, Pengfei Wang, Shuai Zhang, Tingzhen Yang, Huacheng Cai, Qian Wang, Peitao Lü, Jiancheng Zhang

**Affiliations:** ^1^ College of Horticulture, Shanxi Agricultural University, Jinzhong, China; ^2^ College of Horticulture, FAFU-UCR Joint Center for Horticultural Biology and Metabolomics, Haixia Institute of Science and Technology, Fujian Agriculture and Forestry University, Fuzhou, China; ^3^ Fruit Research Institute, Shanxi Agricultural University, Jinzhong, China

**Keywords:** anthocyanin, flavonol, fruit ripening, expression regulation, omics

## Abstract

Fruit ripening is accompanied by a wide range of metabolites and global changes in gene expression that are regulated by various factors. In this study, we investigated the molecular differences in red apple ‘Hongmantang’ fruits at three ripening stages (PS1, PS5 and PS9) through a comprehensive analysis of metabolome, transcriptome and chromatin accessibility. Totally, we identified 341 and 195 differentially accumulated metabolites (DAMs) in comparison I (PS5_vs_PS1) and comparison II (PS9_vs_PS5), including 57 and 23 differentially accumulated flavonoids (DAFs), respectively. Intriguingly, among these DAFs, anthocyanins and flavonols showed opposite patterns of variation, suggesting a possible competition between their biosynthesis. To unveil the underlying mechanisms, RNA-Seq and ATAC-Seq analyses were performed. A total of 852 DEGs significantly enriched in anthocyanin metabolism and 128 differential accessible regions (DARs) significantly enriched by MYB-related motifs were identified as up-regulated in Comparison I but down-regulated in Comparison II. Meanwhile, the 843 DEGs significantly enriched in phenylalanine metabolism and the 364 DARs significantly enriched by bZIP-related motifs showed opposite trends. In addition, four *bZIPs* and 14 *MYBs* were identified as possible hub genes regulating the biosynthesis of flavonols and anthocyanins. Our study will contribute to the understanding of anthocyanins and flavonols biosynthesis competition in red apple fruits during ripening.

## Introduction

The old saying ‘An apple a day keeps the doctor away’ can be explained by the fact that apples can help people to stay healthy and away from the doctor by reducing the risk of numerous chronic diseases due to its high antioxidant content. As a major contributor to the total antioxidant capacity, flavonoids were hence considered as one of the most important phytonutrients that apples nourish people (Lee et al., 2003). Apples are identified as one of the main diet sources of flavanols and flavonols for people (Vrhovsek et al., 2004). Moreover, the anthocyanins accumulation in apples skin and flesh affects greatly the external quality and health care values of apples (Martin, 2013). Therefore, the study of flavonoids, especially anthocyanin biosynthesis, has been an attention hotspot for apple studies.

To date, there are more than 10,000 known flavonoids that have been identified. According to their structures, they can be divided into six groups: dihydroflavones, flavones, isoflavones, flavonols, flavanols and anthocyanidins. The main synthetic pathway of flavonoids are well established, i.e., dihydro-flavonoids are first synthesized, and then flavonoids, isoflavones, flavonols, flavanols and anthocyanidins are further synthesized through a series of branching pathways catalyzed by enzymes encoded by structural genes, such as phenylalanine ammonia-lyase (PAL), cinnamic acid 4-hydroxylase (C4H), 4-coumarate-CoA ligase (4CL), chalcone synthase (CHS), chalcone isomerase (CHI), flavanone-3-hydroxylase (F3H), flavonoid-3’-hydroxylase (F3’H), flavonoid-3’-5’-hydroxylase (F3’5’H), flavonol synthase (FLS), dihydroflavonol reductase (DFR), anthocyanidin synthase (ANS), leucocyanidin reductase (LAR), anthocyanidin reductase (ANR), etc. (Gutierrez et al., 2017). In addition to structural genes, the flavonoids biosynthesis is also regulated by many transcription factors (TFs), which are directly or indirectly modulating the spatiotemporal expression of structural genes (Zhang et al., 2021). Among them, MYB has long been considered as one of the most important TFs regulating the flavonoids synthesis in apple (Takos et al., 2006). Some other TFs have also contributed greatly to the regulation of flavonoids biosynthesis, mainly through interacting with various MYB members. For example, EIL3 (EIN3-LIKE1) can bind to the promoter of *MdMYB1* to activate *MdMYB1* gene expression and to induce anthocyanin accumulation and fruit color transformation (An et al., 2018c); MdERF1B interacts with MdMYB9, MdMYB1 and MdMYB11 and can bind to the promoters of their encoded genes to affect anthocyanin biosynthesis (Zhang et al., 2018).

Recently, integrated metabolomic and transcriptomic analyses have been successfully applied in the exploration of the key factors regulating metabolites accumulations in many fruits (Xu H. et al., 2020; Zhang et al., 2020; Fu et al., 2022). However, the two methods can only reflect changes in gene expression and metabolites accumulation at the RNA and metabolite levels, respectively, and the factors influencing gene expression cannot be directly deciphered. Access to open chromatin can reveal the regulation mechanisms of gene expression, which is a hot topic in epigenetic research. ATAC-seq (Assay for Transpoase-accessible Chromatin with High Throughput sequencing), an innovative technology developed by Buenrostro et al. (2013), is an epigenetic research technique for analyzing Tn5 transposase accessibility chromatin region using high-throughput sequencing and is considered as a potential tool for identifying *cis*-regulatory regions such as TF binding sites (Grandi et al., 2022). Based on the ATAC-Seq data, all the open chromatin regions in a specific spatiotemporal condition could be obtained. Through investigating the accessible chromatin regions (ACRs) in root, leaf bud, flower, flower bud, developing seed, and pod of soybean using ATAC-Seq, Huang et al. (2022) reported that the ACRs occupied more than 3% of the soybean genome. By integrating the RNA-seq and TF ChIP-seq results, they found that ACRs were tightly associated with gene expressions and TF binding capacities. In *Vitis vinifera* leaves, more than 16,000 ACRs were identified, with nearly 5,000 distal enhancer candidates located in intergenic regions > 2 kb from the nearest transcription start site (TSS), which were found to be enriched in TCP family binding sites by motif search, suggesting that these regions may be regulated by TCP (Schwope et al., 2021). Through the combined ATAC-Seq and RNA-Seq analyses, nine TF genes, including *RAV1*, were identified as responsible for high cold hardiness of *V*. *amurensis* (Ren et al., 2021).

Red-flesh apples, containing anthocyanins in both fruit skin and flesh, are popular for their anthocyanin-rich characteristics. The red-flesh apple ‘Hongmantang’, selected from *Malus pumila* Mill. × *M. baccata* (L.) Borkh hybrids, is of purple-red flower and blood-red flesh. Compared with its parent *M. pumila* Mill., its total anthocyanins and total polyphenols contents in fruits are much higher, suggesting that it can be used as a suitable material for the flavonoids biosynthesis studies of *Malus* species (Yang et al., 2015). In our previous study, through analyzing the changes in total anthocyanins contents during fruit ripening, we found that there were two anthocyanin content peaks (at PS5 and PS9, respectively) throughout fruit ripening stage (Guo et al., 2021) (**Figure S1**). In this study, to reveal the mechanisms underlying the bioactive compounds changes during fruit ripening, we investigated the metabolome, transcriptome and chromatin accessibility changes in ‘Hongmantang’ fruits at three ripening stages, PS1 (at about 7 weeks post flowering with the lowest anthocyanin content), PS5 (at about 15 weeks post flowering with the highest anthocyanins content) and PS9 (at about 23 weeks post flowering, the second anthocyanins accumulation peak), by using the ultra-performance liquid chromatography-tandem mass spectrometry (UPLC-MS/MS)-based widely targeted metabolome, RNA-Seq and ATAC-Seq technologies, respectively. The results obtained in this study could provide a basis for the comprehensive understanding of the metabolites changes during fruit ripening in red apple.

## Materials and methods

### Plant materials

The red apple ‘Hongmantang’ used in this study is an ornamental red apple with purple-red flower and blood-red flesh, and was selected from *M. pumila* Mill. × *M. baccata* (L.) Borkh interspecific hybrids by Shanxi Academy of Agricultural Sciences. In this study, the ‘Hongmantang’ fruits were harvested at three key developmental stages (PS1: at about 7 weeks post flowering; PS5: at about 15 weeks post flowering; PS9: at about 23 weeks post flowering) and stored in a -80°C freezer for further use.

### Metabolites extraction and determination

Briefly, 50 mg of red apple fruit samples were added to 1 mL of 70% methanol solution, homogenized at 30 Hz for 3 min, vortexed for 1 min, placed on ice for 15 min, centrifuged at 12,000 rpm at 4°C for 10 min, and then the supernatant was collected for subsequent LC-MS/MS analysis at Wuhan MetWare Biotechnology Co., Ltd. (Wuhan, China). The analysis was performed using a liquid chromatography-electrospray ionization-mass spectrometry (LC-ESI-MS/MS) system according to the method described by Chen et al. (2013). The column of Agilent SB-C18 (1.8 µm, 2.1 mm * 100 mm) and the mobile phase consisted of solvent A (ultrapure water with 0.04% acetic acid) and solvent B (acetonitrile with 0.04% acetic acid) were used in the analysis. The gradient program was set as follows: 95% solvent A and 5% solvent B at 0 min; a liner gradient to 5% solvent A and 95% solvent B within 9 min and kept for 1 min; and adjustment to 95% solvent A and 5% solvent B within 1.1 min and kept for 2.9 min (Li et al., 2022). The flow rate was 0.35 mL/min with injection volume of 4 μL, and the column temperature was 40°C. The effluent was connected to an ESI-triple quadrupole-linear ion trap (QTRAP)-MS (AB4500 Q TRAP UPLC/MS/MS System) equipped with an ESI Turbo Ion-Spray interface. Analyst 1.6.3 software (AB Sciex) was used for controlling the positive and negative ion modes. The MetWare database (MWDB) together with several public databases were used for metabolites annotation (Li et al., 2022). And metabolites were quantified using the multiple reaction monitoring method. Three biological replicates were performed on fruits at each selected stage (PS1, PS5 and PS9). The OPLS-DA (Orthogonal Projection to Latent Structures Discriminant Analysis) model was established using multiple supervision methods and examined using the Variable Importance in Projection (VIP) parameter. For the identification of differentially accumulated metabolites (DAMs) in fruits of the three ripening stages, VIP ≥ 1 and fold change ≥ 2 or ≤ 0.5 were used as criteria (Yuan et al., 2018; Zhang et al., 2020).

### RNA extraction, quantification and transcriptome sequencing

Total RNA of red apple fruits was extracted using Trizol (Invitrogen, CA, USA). After digestion of DNA contamination with DNase I, the RNA quality and quantity were detected using NanoDrop 2000 spectrophotometer (NanoDrop Technologies, Wilmington, DE, USA) and a Bioanalyzer 2100 system (Agilent Technologies, CA, USA). High-quality total RNA was used for RNA-Seq on a MGI-SEQ 2000 platform to generate 150 bp paired-end reads at Wuhan Frasergen Bioinformatics Co., Ltd. (Wuhan, China). The raw clean reads were mapped to the *M. baccata* (L.) Borkh. genome [downloaded from https://www.ncbi.nlm.nih.gov/genome/?term=Malus_baccata_v1.0] for gene transcriptional level studies using hisat2 software. For each stage, three biological replicates were performed, with at least ten fruits being mixed in each replicate. Differentially expressed genes (DEGs) were identified using DESeq2 with criteria of log2(fold change) > 1 or < -1 and corrected *q*-value < 0.05. The RNA-Seq raw data has been deposited at NCBI under the BioProject number PRJNA861071 (https://www.ncbi.nlm.nih.gov/sra/PRJNA861071).

### ATAC-Seq library construction and analysis

ATAC-Seq libraries were constructed and sequenced according to the methods described by Buenrostro et al. (2013) and Corces et al. (2017). For fruits at each stage, two biological replicates were performed. Briefly, total DNA from Tn5 digestion was purified by phenol/chloroform extraction and precipitation, electrophoresed on a 2% agarose gel, amplified using PCR, purified and sequenced by Illumina method. clean reads were mapped to the *M. baccata* genome using Bowtie2 (Langmead and Salzberg, 2012), and redundancy was removed from the obtained comparison files using the Picard program, followed by peak detection by MACS2. The distribution of peaks in each sample relative to gene transcription start sites (TSSs) and transcription end sites (TESs) was statistically analyzed and the proportion of peaks at various locations in the genome, including 5’UTR, 3’UTR, 1^st^ exon, other exon, 1^st^ intron, other intron, downstream (≤ 300 bp) and distal intergenic, was calculated. For the identification of differential peaks, DESeq2 was used with a threshold of FDR (false discovery rate) < 0.05 and fold change > 1.5 or < -1.5. The GenomicFeatures (R packages) was used to extract the genes nearby the differential peaks. For the enrichment analysis of genes involved in differential accessible regions (DARs), GO and KEGG enrichment analyses were performed with *P* (classicFisher) ≤ 0.05 and corrected *P*-value < 0.05, respectively. The ATAC-Seq raw data has been deposited at NCBI under BioProject number PRJNA860789 (https://www.ncbi.nlm.nih.gov/sra/PRJNA860789).

### Quantitative real time PCR analysis

Complementary DNA (cDNA) of different samples was synthesized separately using the PrimeScript RT Master Mix (Perfect Real Time) kit (Takara, Dalian, China). Gene specific primers for selected genes were designed using Primer 3 (https://bioinfo.ut.ee/primer3-0.4.0/) according to their CDS (coding sequence) sequences (**Table S1**). qRT-PCR experiment was performed on Efficiency T96 real-time quantitative fluorescent PCR instrument (Beijing Leadaeon Technology Co. Ltd, Beijing, China). The qRT-PCR conditions were set as follows: pre-denaturation at 95°C for 30 s; denaturation at 95°C for 10 s, annealing at 54.8~58.8°C for 20 s, extension at 72°C for 20 s, 50 cycles. qRT-PCR reaction system consisted of 10 µL SYBR Premix ExTaq™ (TaKaRa) fluorescent dye (containing Oligo (dT) and random primers), 7.4 µL ddH_2_O, 0.8 µL of each upstream and downstream primer, and 1 µL cDNA template. Their relative expression levels in different samples were calculated using the 2^−ΔΔCt^ method with *actin* as internal reference gene (Jiang et al., 2019).

## Results

### UPLC-MS/MS analysis of metabolic profiles in red apple fruits during ripening

The UPLC-MS/MS-based untargeted metabolome approach enables the detection of many primary and secondary metabolic compounds from red apple fruit samples. Totally, we identified 753 metabolites from red apple fruits at three ripening stages, including 399 primary metabolites and 354 secondary metabolites (**Table S2**). These metabolites could be further classified into 11 groups, including phenolic acids, flavonoids, lipids, amino acids and derivatives, organic acids, nucleotides and derivatives, alkaloids, terpenoids, tannins, lignans and coumarins, and others (**Figure S2**). Metabolites belonging to amino acids and derivatives, flavonoids, alkaloids, phenolic acids, nucleotides and derivatives, lignans and coumarins, and tannins mostly showed high accumulation at PS1 but low accumulation at PS5. However, many terpenoids and some lipids and phenolic acids accumulated highly at PS9. Moreover, some metabolites, such as saccharides and alcohols, vitamin and stilbene, were classified into ‘others’ group and showed very low or even no accumulation at PS1, but their contents increased with fruit ripening.

**Figure 1:**
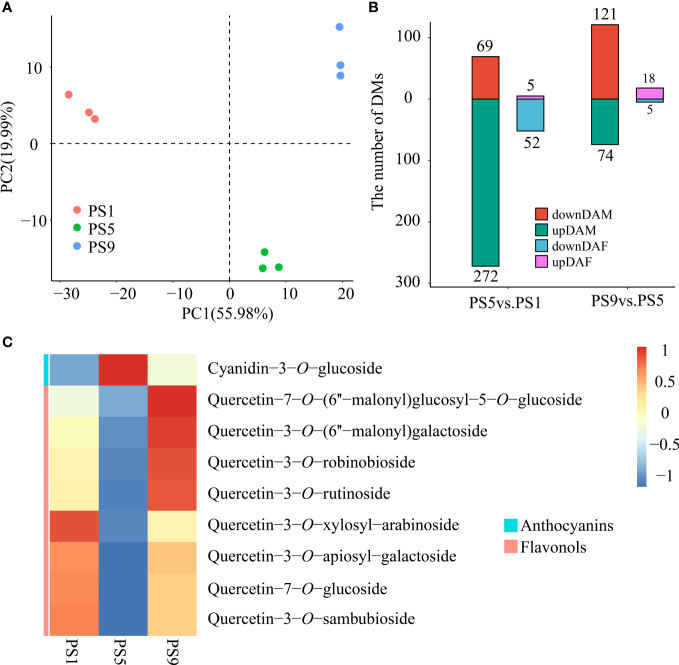
Metabolome differences in red apple fruits at three different ripening stages. **(A)** PCA analysis of red apple fruit metabolome data at PS1, PS5 and PS9; **(B)** The numbers of differentially accumulated metabolites and flavonoids in two comparisons. DAM, differentially accumulated metabolites; DAF, differentially accumulated flavonoids; **(C)** Differential accumulation was commonly identified in comparisons of PS5_vs_PS1 and PS9_vs_PS5 of anthocyanins and flavonols.

PCA analysis of the metabolites among red apple fruits at three different ripening stages revealed that their metabolomes were clearly different (**Figure 1A**). A total of 341 differentially accumulated metabolites (DAMs) were identified, including 69 increased and 272 decreased DAMs in comparison PS5_vs_PS1 (Comparison I) (**Table S3**; **Figure 1B**). There were much less increased metabolites than decreased metabolites for DAMs belonging to phenolic acids (16 increased and 54 decreased), flavonoids (5 increased and 52 decreased), lipids (7 increased and 41 decreased), amino acids and derivatives (4 increased and 37 decreased), organic acids (9 increased and 16 decreased), alkaloids (3 increased and 16 decreased), terpenoids (1 increased and 13 decreased), nucleotides and derivatives (2 increased and 9 decreased), tannins (only 9 decreased) and lignans and coumarins (2 increased and 5 decreased). However, for the saccharides and alcohols related DAMs, a larger proportion of increased metabolites (17 increased and 14 decreased) was observed. Notably, among the 57 different accumulated flavonoids (DAFs) (**Table S4**; **Figure 1B**), two anthocyanins, cyanidin-3*-O*-galactoside and cyanidin-3*-O*-glucoside (Kuromanin), increased in PS5 for 1.57- and 2.31-fold, respectively.

A total of 195 DAMs were identified in PS9_vs_PS5 (Comparison II), including 121 increased and 74 decreased (**Table S5**). There were more increased than decreased metabolites for DAMs belonging to phenolic acids (24 increased and 6 decreased), flavonoids (18 increased and 5 decreased), lipids (23 increased and 8 decreased), terpenoids (24 increased and 2 decreased) and lignans and coumarins (5 increased and 2 decreased). However, more metabolites were decreased than increased for DAMs belonging to amino acids and derivatives (6 increased and 19 decreased), organic acids (8 increased and 9 decreased), alkaloids (1 increased and 6 decreased), nucleotides and derivatives (4 increased and 7 decreased) and tannins (only 1 decreased).

For the DAMs belonging to amino acids and derivatives, organic acids, alkaloids, nucleotides and derivatives and tannins, the number of decreased DAMs was higher than that of the increased ones in both comparisons, suggesting that the accumulations of these kinds of metabolites mostly reduced as fruit ripened. In contrast, the number of the increased saccharides and alcohols related metabolites was both higher than that of the decreased ones, which is in consistent with the sugar accumulation during fruit ripening. Moreover, the numbers of increased DAMs belonging to phenolic acids, flavonoids, lipids, terpenoids, and lignans and coumarins were much lower than the that of the decreased ones in Comparison I, but much higher than the numbers of decreased ones in Comparison II, suggesting that the accumulation of these kinds of metabolites reduced in the early fruit development stages but increased in the late fruit ripening stages.

Interestingly, we found that in Comparison I, all differentially accumulated anthocyanins increased, while all differentially accumulated quercetins decreased (**Table S4**). In Comparison II, we found a decrease in all differentially accumulated anthocyanins (**Table S6**), and an increase in all quercetins except quercetin-3*-O*-rhamnosyl (1→2) arabinoside (**Table S6**). In both comparisons, Cyanidin-3*-O*-glucoside and the eight quercetins were found to be DAMs, and showed completely opposite expression trends (**Figure 1C**). Therefore, we conclude that there is dynamic competition between anthocyanins and flavonols biosynthesis during red apple fruit development and ripening.

### Comparative transcriptome analysis results

**Figure 2:**
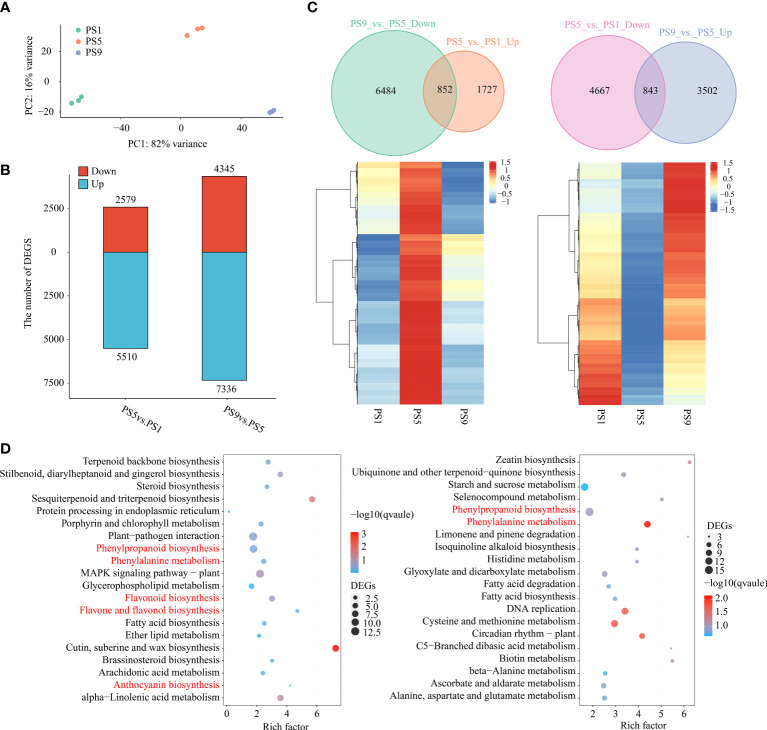
Comparative transcriptome analysis of red apple fruits at three different ripening stages. **(A)** PCA analysis result of red apple transcriptome data for PS1, PS5 and PS9; **(B)** Number of differentially expressed genes; **(C)** Venn diagram and heatmap of DEGs up-regulated in PS5_vs_PS1 but down-regulated in PS9_vs_PS5 (left) and DEGs down-regulated in PS5_vs_PS1 but up-regulated in PS9_vs_PS5 (right); **(D)** KEGG enrichment analysis of DEGs showing the same pattern of changes in anthocyanins (left) and flavonols (right) accumulations during fruit ripening.

To further explore the molecular mechanisms regulating the dynamic of anthocyanins and flavonols, especially quercetins, during red apple fruit ripening, we performed RNA-seq analysis on red apple fruits at PS1, PS5 and PS9 stages. PCA analysis of transcriptome data showed that red apple fruits at the three ripening stages were well distinguished from each other (**Figure 2A**). The mapping ratios of all transcriptome data from the nine cDNA libraries against the reference genome were all above 79% (**Table S7**). By using |log2 (Fold change)| ≥ 1 and *q*-value ≤ 0.05 as criteria, we identified 8,089 differentially expressed genes (DEGs, 2,579 up-regulated and 5,510 down-regulated) in PS5_vs_PS1, and 11,681 DEGs (4,345 up-regulated and 7,336 down-regulated) in PS9_vs_PS5 (**Figure 2B**). Among them, 852 DEGs were up-regulated in PS5_vs_PS1 but down-regulated in PS9_vs_PS5 (**Figure 2C**; **Table S8**), which showed the same dynamic patterns as anthocyanins accumulation during fruit ripening. KEGG enrichment analysis showed that these 852 common DEGs were involved in phenylpropanoid biosynthesis, phenylalanine metabolism, flavonoid biosynthesis, flavone and flavonol biosynthesis and anthocyanin biosynthesis pathways (**Figure 2D**). Moreover, we identified 843 DEGs up-regulated in PS9_vs_PS5 but down-regulated in PS5_vs_PS1 (showing the same dynamic patterns as most flavonols, especially quercetins) (**Figure 2C**; **Table S9**). KEGG enrichment analysis showed that these genes were significantly enriched in phenylalanine metabolism, phenylpropanoid biosynthesis, etc. (**Figure 2D**). Among the 852 DEGs that were found to be up-regulated in PS5_vs_PS1 but down-regulated in PS9_vs_PS5 and the 843 DEGs up-regulated in PS9_vs_PS5 but down-regulated in PS5_vs_PS1, there were many flavonoid biosynthesis structural genes, including seven *PALs*, fifteen *4CLs*, two *CHSs*, four *CHIs*, three *F3Hs*, three *F3’5’Hs*, five *F3’H*s, four *FLSs*, two *DFRs*, one *ANS*, three *LARs*, four *ANRs*, and two *UFGTs* (**Figure S3**). Interestingly, almost all the DEGs encoding enzymes catalyzing the reactions from dihydroflavonols to anthocyanidins, such as *F3’5’H*, *F3’H*, *DFR* and *ANS*, showed the lowest expression at PS9, whereas the two genes encoding FLS enzyme catalyzing the conversion of dihydroflavonols to flavonols showed the highest expression at PS9.

Quantitative real time PCR (qRT-PCR) results showed that all the expression patterns of our selected flavonoids biosynthetic structural genes were identical to our transcriptome data, suggesting that our transcriptome data are reliable. The lowest expression of *DFR* was also found at PS9, indicating that its low expression might be closely related to the anthocyanins reduction at this stage. The *FLS* gene, however, showed the lowest expression at PS5 but the highest expression at PS9 according to our transcriptome and qRT-PCR results, which was the same pattern as the change in flavonols content (**Figure S4**).

### Chromatin accessibility dynamics in red apple fruits during ripening

**Figure 3:**
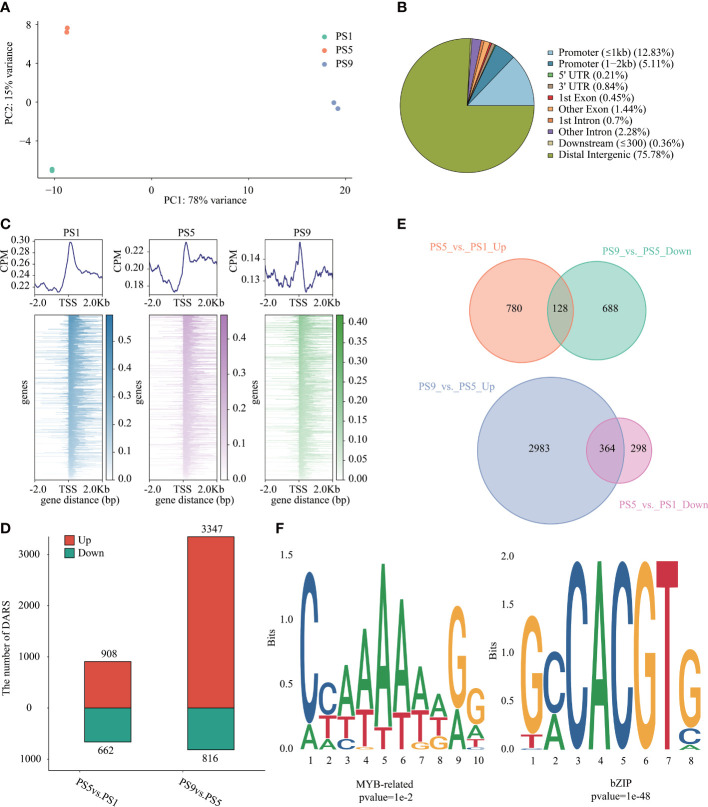
Chromatin accessibility features of red apple fruit during ripening. **(A)** PCA analysis result of red apple ATAC-Seq data at PS1, PS5 and PS9; **(B)** Relative location of the identified peaks in different gene regions; **(C)** Relative location of peaks to red apple genes in the upstream of 2 Kb and downstream of 2 Kb in the TSSs region; **(D)** Numbers of DARs in different comparisons; **(E)** Venn diagrams of DARs up-regulated in PS5_vs_PS1 but down-regulated in PS9_vs_PS5 (top) and DARs down-regulated in PS5_vs_PS1 but up-regulated in PS9_vs_PS5 (bottom); **(F)** Motif enrichment analysis results of MYB related motifs in DARs up-regulated in PS5_vs_PS1 but down-regulated in PS9_vs_PS5 and bZIP motifs in DARs up-regulated in PS9_vs_PS5 but down-regulated in PS5_vs_PS1.

ATAC-Seq was applied to investigate the chromatin accessibility changes during ripening in red apple (**Table S10**). PCA analysis of the ATAC-Seq data showed a clear distinction in chromatin accessibility among the three ripening stages in red apple fruits (**Figure 3A**). We identified 41,292 to 58,869 peaks in each ATAC-Seq library (**Table S11**; **Figure S5**). Relative location analysis of peaks showed that they were mainly distributed in distal intergenic region (75.78%), promoter (≤ 1 kb) region (12.83%) and promoter (1-2 kb) region (5.11%) (**Figure 3B**), with their distributions peaked around the TSSs (**Figure 3C**). By using FDR < 0.05 and FC > 1.5 or FC < -1.5 as criteria, we identified 908 up-regulated differential accessible regions (DARs) and 662 down-regulated DARs in PS5_vs_PS1, and 3,347 up-regulated and 816 down-regulated DARs in PS9_vs_PS5 (**Figure 3D**, **Table S12**).

To show the possible regulation of chromatin accessibility on the expression of genes related to anthocyanins and flavonols biosynthesis, we further focused on DARs showing opposite patterns of change between PS5_vs_PS1 and PS9_vs_PS5. In total, we identified 128 DARs up-regulated in the former comparison but down-regulated in the latter one (consistent with the pattern of change for differentially accumulated anthocyanins) (**Figure 3E**, **Table S13**), and 364 DARs up-regulated in the later comparison but down-regulated in the former one (consistent with the pattern of change for differentially accumulated quercetins) (**Figure 3E**, **Table S14**). The MYB-bHLH-WD40 (MBW) regulatory complex has been frequently discovered to function in regulating flavonoids biosynthesis (Zhao et al., 2019). Among the DARs up-regulated in PS5_vs_PS1 and down-regulated in PS9_vs_PS5, we identified two *MYB* genes (C1H46_025763 and C1H46_027060) and one *bHLH* gene (C1H46_000912) (**Figure S6A**). Moreover, ten *MYB* genes, eleven *bHLH* genes and one *WD40* gene were found to be down-regulated in the DARs of PS5_vs_PS1 and up-regulated in PS9_vs_PS5 (**Figure S6B**). Interestingly, two *bHLH* genes (C1H46_035986 and C1H46_021331) are identified in the common DARs. A motif enrichment analysis using HOMER showed that MYB-related motifs were significantly enriched in the DARs with the same change pattern as the differentially accumulated anthocyanins (**Figure 3F**, **Table S15**), while bZIP-related motifs were significantly enriched in the DARs with the same change pattern as differentially accumulated flavonols (**Figure 3F**, **Table S16**).

**Figure 4:**
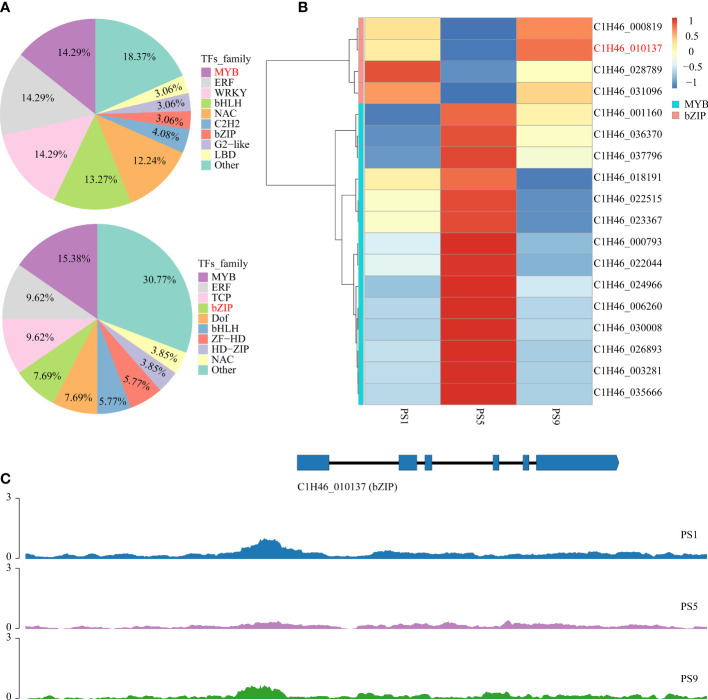
Candidate TF genes that play roles in the competition for anthocyanins and quercetins biosynthesis. **(A)** Frequencies of the transcription factor gene families in all the differentially expressed transcription factor genes; **(B)** Heatmap of differentially expressed *MYB* and *bZIP* genes that showed adverse change patterns in comparison PS5_vs_PS1 and comparison PS9_vs_PS5. **(C)** Chromatin accessibilities of a *bZIP* gene (C1H46_010137) at three different ripening stages.

To further reveal the potential regulatory roles of TFs on the expression of genes related to anthocyanins and flavonols biosynthesis, PlantTFDB was applied to predict the genes encoding TFs in the above common DEGs. The results showed that among the 852 common DEGs that were up-regulated in PS5_vs_PS1 but down-regulated in PS9_vs_PS5, genes encoding MYB, ERF, WRKY, bHLH and NAC accounted for more than 10% of all TF genes, with the percentages of 14.29%, 14.29%, 14.29%, 13.27% and 12.24%, respectively (**Figure 4A**; **Table S17**). In contrast, among the 843 common DEGs up-regulated in PS9_vs_PS5 but down-regulated in PS5_vs_PS1, genes encoding TF family proteins such as MYB, ERF, TCP, bZIP, Dof, bHLH and ZF-LD accounted for a larger proportion of all TF-encoding DEGs, accounting for 15.38%, 9.62%, 9.62%, 7.69%, 7.69%, 5.77% and 5.77%, respectively (**Figure 4A**; **Table S18**).

By integrating the results of metabolome and motif enrichment analysis, it was predicted that TF MYBs have many functions and different MYB members play various roles in regulating both anthocyanins and flavonols biosynthesis, while bZIP transcription factors might play important roles in regulating flavonols biosynthesis during fruit ripening in red apple. Among these *MYB* and *bZIP* genes, four *bZIPs* and 14 *MYBs* showed significant positive correlations with anthocyanins and quercetins contents, respectively (**Figure 4B**; **Table 1**), and they could be considered as hub genes regulating the dynamics of anthocyanins and quercetins, respectively. Among these *bZIP* and *MYB* genes, some of their homologous genes, including Arabidopsis *vip1*, pear *MYB10*, apple *MYB24-like* and *MYB11* have been reported to be associated with anthocyanin biosynthesis (Stracke et al., 2001; Sun et al., 2019; Wang et al., 2019a; Li et al., 2020b; Land et al., 2021). Notably, the chromatin accessibility in the promoter region of one *bZIP* gene (C1H46_010137) was found to be positively correlated with its gene expression level at the three stages (**Figure 4C**; **Tables S19**, **S20**), and its function in the competition for anthocyanins and flavonols biosynthesis needs to be further explored in future studies. Moreover, our qRT-PCR results showed that the expression patterns of the two selected *MYB* and three *bZIP* genes were identical to our transcriptome data (**Figure S4**), which well supported our transcriptome data and indicated that these TFs might contribute greatly to the competition between anthocyanins and flavonols biosynthesis.

**Table 1:** The identified differentially expressed *bZIP* and *MYB* genes that showed opposite change patterns between PS5_vs_PS1 and PS9_vs_PS5 comparisons.

TF family	Gene ID	Tophit homologous genes/similarity	Homologous Arabidopsis gene/similarity	References
bZIP	C1H46_000819	apple VIP1-like (XM_008340896)/55.17%	AT1G43700 (SUE3,VIP1)/43.81%	Land et al., 2021
	C1H46_010137	apple transcriptional activator hacA-like (XM_008369083.3)/66.95%	AT1G19490 (bZIP62)/49.27%	
	C1H46_028789	apple basic leucine zipper 43-like (XM_008395159)/66.88%	AT5G60830 (bZIP70)/46.73%	
	C1H46_031096	apple transcriptional activator hacA-like (XM_017331100)/54.57%	AT1G19490 (bZIP62)/45.14%	
MYB	C1H46_000793	apple MYB30-like (XM_008366038.3)/71.03%	AT2G31180 (MYB14)/56.61%	
	C1H46_001160	apple MYB55 (MG099798)/86.61%	AT5G57620 (MYB36)/53.73%	
	C1H46_003281	apple MYB93-like (XM_008347508)/81.80%	AT1G34670 (MYB93)/57.22%	
	C1H46_022044	apple MYB25 (NM_001293983.1)/67.28%	AT2G31180 (MYB14)/51.8%	
	C1H46_023367	pear MYB10 (KT601121.1)/96.33%	AT1G56650 (MYB7)/52.62%	Li X. et al., 2020
	C1H46_024966	apple MYB93-like (XM_008385715.3)/76.39%	AT1G34670 (MYB93)/58.06%	
	C1H46_026893	apple MYB24-like (XM_029096516.1)/55.08%	AT4G13480 (MYB79)/51.7%	Wang et al., 2019a
	C1H46_030008	apple MYBR24 (MG099810.1)/89.46%	AT2G38090/61.65%	
	C1H46_036370	apple WER-like (XM_008341803.3)/69.74%	AT5G40330 (MYB23)/54.46%	
	C1H46_037796	apple MYB8-like (XM_008347850.3)/84.64%	AT3G12720 (MYB67,ATY53)/49.31%	
	C1H46_018191	apple MYB11 (NM_001294029.1)/50.48%	AT5G35550 (MYB123)/36.27%	Stracke et al., 2001; Sun set al., 2019
	C1H46_022515	apple ETC1 (XM_008347609.3)/31.30%	AT4G01060 (ETC3)/51.01%	
	C1H46_006260	apple MYB42 (MG099785.1)/64.68%	AT4G21440 (MYB102)/40.23%	
	C1H46_035666	apple MYB42 (MG099785.1)/60.09%	AT3G12720 (MYB67,ATY53)/26.7%	

## Discussion

Fruit ripening is a complex process accompanied by extensive metabolites and global changes in gene expression controlled by a variety of factors, including developmental and environmental factors. These changes varied greatly in different plant species and different cultivars of the same plant species. *Malus* spp. is rich in flavonoids, especially in their flowers and fruits. In this study, we investigated the metabolome, transcriptome and chromatin accessibility changes of apple ‘Hongmantang’ with purple-red flower and blood-red flesh during fruit ripening, through integrative analyses of the datasets of UPLC-MS/MS-based widely targeted metabolome, RNA-Seq and ATAC-Seq from three ripening stages (PS1, PS5 and PS9).

### Changes in the accumulation of different kinds of metabolites varied markedly during red apple fruit ripening

In the study by Xu et al. (2020), more than half of the metabolites identified in apple (*M. domestica*) fruits showed high accumulation at early ripening stages. In our present study, we found that most metabolites belonging to amino acids and derivatives, organic acids, nucleotides and derivatives and tannins accumulated the highest in red apple fruits at PS1, and the numbers of decreased DAMs were both higher than that of the increased DAMs in the two comparisons, suggesting that the accumulations of these kinds of metabolites mostly reduced as fruit ripened. However, the numbers of the increased saccharides and alcohols related metabolites were both higher than the decreased ones, which is in consistent with the sugar accumulation during fruit ripening (Xu et al., 2020). Moreover, the numbers of increased DAMs belonging to phenolic acids, flavonoids, lipids, terpenoids, lignans and coumarins were much lower than the decreased ones in the comparison PS5_vs_PS1, but were much higher than the increased ones in the comparison PS9_vs_PS5, suggesting that the accumulations of these kinds of metabolites are associated with the red apple ripening process.

Given that they share the same biosynthesis substrates, competition occurred between anthocyanins and flavonols biosynthesis pathways in many plants (Luo et al., 2016; Zhong et al., 2020; Wong et al., 2022). For example, a comparison of anthocyanins and flavonols contents in the petals of ten *Rhododendron* species revealed that species with low anthocyanin contents had high flavonols (Liu et al., 2016); in *Malus*, competition between anthocyanins and kaempferol glycosides was found to be related to pollen tube growth and seed set (Chen et al., 2021); in bilberry, procyanidins and quercetins were identified as the major flavonoids in the early fruit ripening stage, but anthocyanins accounted for the largest fraction in the later ripening stages (Jaakola et al., 2002). In our present study, we found that many flavonoids were identified as DAMs during red apple fruit ripening. Moreover, in both PS5_vs_PS1 and PS9_vs_PS5 comparisons, cyanidin-3*-O*-glucoside and eight quercetins were found to be DAMs, but showed completely opposite accumulation trends. Therefore, it is hypothesized that there is a dynamic competition between anthocyanins and flavonols biosynthesis in red apple fruits during fruit development and ripening.

### The differential expression of structural genes for flavonoids biosynthesis contributed greatly to the accumulations of different kinds of flavonoids

The accumulations of anthocyanins and flavonols largely contribute to the color changes during fruit ripening (Jaakola et al., 2002). Dihydroflavonols, the intermediates and branch point of the anthocyanins and flavonols biosynthetic pathways, are required for anthocyanins and flavonols productions and as substrates for their competition (Davies et al., 2003). This competitive process is controlled by many biosynthetic genes, especially *DFRs* and *FLSs* (Chen et al., 2021). In tomato, the *anthocyanin without* mutant, harboring a defect in *DFR*, displayed elevated flavonols and anthocyanins deficiency, and another mutant, *anthocyanin reduced* (*are*) has been reported to be caused by the mutation in a *FLS*, a gene encoding an enzyme catalyzing the first step of flavonol biosynthesis (Maloney et al., 2014). By using the white-flowered, flavonol accumulating Mitchell line of petunia as material, Davies et al. (2003) found that overexpression both of 35SCaMV-*DFR* sense and/or *FLS* antisense transgene led to the anthocyanins production and resulted in a pink-flowered phenotype. Constitutive overexpression of *FLS* genes from *Rosa rugosa*, *Prunus persica*, and *Petunia hybrida* in tobacco all resulted in white flowers, while the heterologous overexpression of *DFR* genes resulted in increased anthocyanins accumulations and redder flowers (Luo et al., 2016; Wong et al., 2022). Liu et al. (2019b) reported that heterologous expression of the *Muscari aucheri MaFLS* gene in tobacco increased *NtFLS* expression, but inhibited the expression of tobacco anthocyanins biosynthesis genes, such as *NtDFR*, *NtANS* and *NtAN2*, and reduced the pigmentation of tobacco petals. Similarly, Park et al. (2020) reported that the overexpression of the onion *FLS* gene (*AcFLS-HRB*) increased the flavonols contents in tobacco petals but decreased the anthocyanins accumulation and produced lighter-pink flowers. In our study, almost all the DEGs encoding enzymes that catalyze the reactions from dihydroflavonols to anthocyanidins were least expressed at PS9, which is in consistent with the relatively low accumulation of anthocyanins in red apple fruits at this stage. Moreover, two red apple *FLS* genes had the highest expression at PS9, which well explains the high accumulation of flavonols in red apple fruit at the late stage. It is therefore concluded that the differential expression of these structural genes contributes greatly to the accumulations of different kinds of flavonoids, and that altering their expression by genetic methods may have great potential for balancing anthocyanins and flavonols production (Davies et al., 2003).

### MYB and bZIP transcription factors played dominant regulatory roles in balancing the biosynthesis of anthocyanins and flavonols

In addition to structural genes, the biosynthesis of flavonoids is also controlled by many TFs (Martins et al., 2013; Zhang et al., 2021). The regulatory roles of MBW complex (consisted of MYB, bHLH and WD40 TFs) in flavonoid biosynthesis are well known (Zhao et al., 2019). Consistently, we identified many *MBW* genes, especially *MYB* and *bHLH* genes, as DEGs during ripening in red apple, indicating that these TFs play important roles in flavonoid biosynthesis in red apple. Moreover, we identified more than twenty *MBW* genes in the DARs that showed opposite change patterns between PS5_vs_PS1 and PS9_vs_PS5 comparisons. For example, the mutant of Arabidopsis *vip1*, a homologous gene of the *bZIP* (C1H46_000819), showed much less anthocyanin accumulation under phosphate-limiting conditions (Land et al., 2021); the pear *MYB10*, a homologous gene of the red apple *MYB* (C1H46_023367) has been proved to play roles in regulating anthocyanin biosynthesis and transport in red-skinned pears (Li et al., 2020); and the apple *MYB24-like* (homologous to red apple *MYB* (C1H46_030008)) and *MYB11* (homologous to red apple *MYB* (C1H46_01819)) genes, were both proved to be involved in flavonoids biosynthesis (Sun et al., 2019; Wang et al., 2019a). As one of the most important TFs regulating the flavonoids synthesis, the regulatory roles of MYBs in both flavonols and anthocyanins biosynthesis have been continuously demonstrated in many fruit trees, such as apple (Takos et al., 2006; An et al., 2018a; An et al., 2018b; Wang et al., 2019a; Wang et al., 2019b; Jia et al., 2020; Xu H. et al., 2020), pear (Li et al., 2020a; Li et al., 2020), grape (Deluc et al., 2006), kiwifruit (Wang et al., 2019; Li et al., 2020a) and blueberry (Han et al., 2021) and so on. These reports showed that MYBs can be extensively involved in regulating flavonoids biosynthesis by interacting with some other TFs and with structural genes of flavonoids biosynthesis, thus affecting the color formation and development of plant organs such as leaves, petals and fruits.

Moreover, there are evidences that MYBs paly diverse roles in regulating flavonols and anthocyanins biosynthesis. For example, overexpression of apple *MdMYB10* gene increased the anthocyanins accumulation but decreased the kaempferol 3*-O*-glycosides contents in flowers (Chen et al., 2021). Overexpression of a *Gerbera hybrida* R2R3-MYB transcription factor gene (*GhMYB1a*) resulted in decreased anthocyanins but increased flavonols accumulations in both gerbera and tobacco (Zhong et al., 2020). In our study, genes encoding MYB transcription factors were found to account for the largest fraction of differentially expressed *TFs*, which showed positive or negative expression patterns with the accumulations of anthocyanins and flavonols. Furthermore, ATAC-Seq analysis revealed that MYB-related motifs were significantly enriched in DARs, with the same change pattern as anthocyanins, which again indicates that MYBs contributed greatly to the regulation of both anthocyanins and flavonols biosynthesis in red apple.

Some other TFs also contribute greatly to the biosynthesis of flavonoids in plant, mainly through interacting with MYBs. Similarly, in our study, we also identified many other *TF* genes that showed positive or negative expression patterns with anthocyanins and flavonols accumulations, such as *ERFs*, *bHLHs*, *WRKYs*, *NACs*, *bZIPs*, *TCPs* and so on. And there were many evidences for the roles of these TFs in flavonoids biosynthesis (An et al., 2018c; An et al., 2020; Li C. et al., 2020; Li et al., 2020b; Ma et al., 2021; Zhang et al., 2021; Tu et al., 2022). Notably, bZIP-related motifs were identified to be significantly enriched in DARs that showed the same change pattern with differentially accumulated anthocyanins, suggesting that such TFs also play a major role in regulating flavonoids biosynthesis in red apple. In consistent with our study, numerous evidences demonstrate that the combinatorial action of bZIP and MYB in controlling the flavonoids biosynthesis in many plants (Stracke et al., 2010; Shin et al., 2013; Zhao et al., 2021). In Arabidopsis, bZIP TFs were shown to be functional in promoting anthocyanins accumulation by interacting with MYBL2 (Nguyen et al., 2015), PFG1/MYB12 (Stracke et al., 2010) and MYB75 (Shin et al., 2013) TFs and anthocyanin biosynthetic genes. In soybean, GmMYB and GmbZIP5 interact synergistically in controlling flavonoids biosynthesis. In tea plants, the CsbZIP1-CsMYB12 interaction mediates flavonols production *via* a coordinated activator-repressor network (Zhao et al., 2021).

Notably, most of the *bZIP* family genes reported to be involved in the regulation of anthocyanins biosynthesis were identified as positive regulators of anthocyanins biosynthesis (Han et al., 2021; Tu et al., 2022), which is also consistent with our results. Arabidopsis HY5 promotes anthocyanin accumulation by upregulating the expression of the *DFR* gene (Zhang et al., 2011). Tomato HY5 has been reported to be functional in mediating the CRY1a-induced anthocyanins biosynthesis (Liu et al., 2018; Qiu et al., 2019), and *SlHY5* frameshift mutation showed decreased anthocyanin accumulation (Qiu et al., 2019). Soybean bZIP TF G/HBF-1 plays a key role in increasing phytoalexin accumulation by binding to the promoter of *CHS* gene (Dröge-Laser et al., 1997). The pear bZIP transcription factor PybZIPa has the ability to bind to the promoter of *PyUFGT* and functions in promoting the light-induced anthocyanin accumulation (Liu et al., 2019a). Grapevine VvHY5 promotes flavonol biosynthesis by activating the expression of *FLS* (Loyola et al., 2016); another grapevine bZIP, VvibZIPC22, can promote the accumulation of several kinds of flavonoids by regulating the expression of the *CHS* and *FLS* genes; and VvHY5 promotes flavonol biosynthesis by activating the expression of an *FLS* gene (Malacarne et al., 2016). In the study by Wang et al. (2022), two pomegranate *bZIP* genes, *PgbZIP16* and *PgbZIP34*, which are associated with anthocyanin biosynthesis, were expressed at much higher levels in red petals than in white petals, and their heterologous transient overexpression in tobacco leaves both resulted in increased anthocyanin accumulation. In apple, An et al. (2018d) reported that the ABA inducible MdbZIP44 functioned in the ABA-induced anthocyanin accumulation by enhancing the binding activity of MdMYB1 to the promoters of anthocyanin biosynthetic genes. All these reports suggest that the *bZIP* TFs identified in our present study might play important roles in regulating the biosynthesis of flavonoids in red apple.

## Conclusions

**Figure 5:**
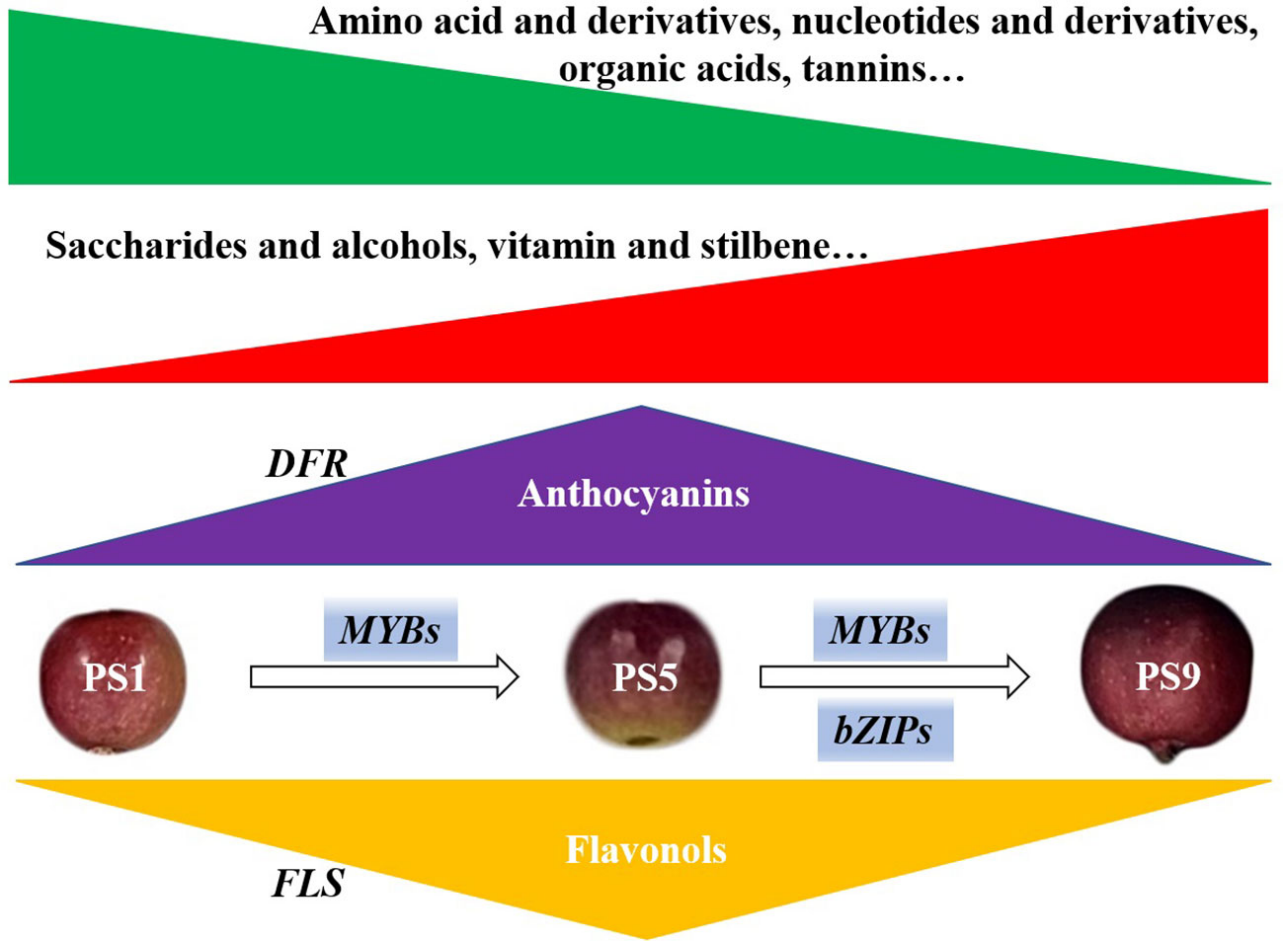
Schematic diagram of the molecular changes in red apple fruits during ripening. The green and red triangle represent the decreased and increased change pattern of metabolites. The accumulations of metabolites mostly belonging to amino acids and derivatives, organic acids, nucleotides and derivatives, and tannins reduced with fruit ripening, while the accumulations of metabolites mostly belonging to saccharides and alcohols, vitamins and stilbene increased with fruit ripening. The accumulation of anthocyanins peaked at PS5 and decreased at PS9. However, the flavonols accumulated least at PS5 but increased at PS9. The differential expression of *DFRs* and *FLSs*, functioned greatly to the accumulations of anthocyanins and flavonols, respectively. Changes in chromatin accessibility and expression of *MYBs* and *bZIPs* suggest that they play dominant regulatory roles in the competition for anthocyanins and flavonols biosynthesis.

In summary, through combined analyses of metabolome, transcriptome and chromatin accessibility datasets, we investigated the molecular changes in red apple ‘Hongmantang’ fruits at three ripening stages (PS1, PS5 and PS9) (**Figure 5**). Metabolic analysis showed that most of the metabolites belonging to amino acids and derivatives, organic acids, nucleotides and their derivatives, and tannins decreased, while most metabolites belonging to saccharides and alcohols, vitamins and stilbene increased with fruit ripening. Notably, a dynamic competition between anthocyanins and flavonols biosynthesis was found. The differential expression of structural genes for flavonoids biosynthesis, such as *FLSs* and *DFRs*, played a major role in the accumulations of the two kinds of flavonoids. Moreover, chromatin accessibility and changes in gene expression of *MYB* and *bZIP* genes were predicted to play dominant regulatory roles in balancing the anthocyanins and flavonols biosynthesis.

## Data availability statement

The original contributions presented in the study are publicly available. This data can be found here: NCBI, PRJNA860789 & PRJNA861071.

## Author contributions

JZ, PL and CC conceived and designed the experiments. CC, ZG, HL, JZ and PL wrote the original draft. CC, ZG, HL, XM, PW, SZ, TY, HC and QW performed the experiments. CC, ZG, HL and PL analyzed the results. CC, HL, XM, PL and JZ reviewed and edited the manuscript. All authors read and approved the final manuscript. The authors have no conflict of interest to declare.

## Funding

This work was supported by the National Key Research and Development Program of China (2018YFD1000200), the earmarked fund for Modern Agro-industry Technology Research System of Shanxi Province, the Reward Fund for PhDs and Postdoctors of Shanxi Province (SXBYKY2022004), the Fund for High-level Talents of Shanxi Agricultural University (2021XG010).

## Conflict of interest

The authors declare that the research was conducted in the absence of any commercial or financial relationships that could be construed as a potential conflict of interest.

## Publisher’s note

All claims expressed in this article are solely those of the authors and do not necessarily represent those of their affiliated organizations, or those of the publisher, the editors and the reviewers. Any product that may be evaluated in this article, or claim that may be made by its manufacturer, is not guaranteed or endorsed by the publisher.

## Supplementary material

Supplementary Figure 1 Changes in phenotype **(A)** and anthocyanin accumulation **(B)** of red apple during fruit ripening. PS1 to P10 represent fruits collected at 7, 9, 11, 13, 15, 17, 19, 21, 23 and 25 weeks post flowering, respectively. There were two anthocyanins accumulation peaks in red apple fruits during ripening, i.e., at PS5 (15 weeks post flowering) and PS9 (23 weeks post flowering), respectively.

Supplementary Figure 2 Heatmap of clustering of metabolites in red apple fruits at three ripening stages. PS1, PS5 and P9 represent fruits collected at 7, 15 and 23 weeks post flowering, respectively. For each stage, three replicates were performed. Red and green colors represent high and low accumulations, respectively.

Supplementary Figure 3 Differentially expressed flavonoids biosynthesis-related genes in red apple fruits at different ripening stages. PAL, phenylalanine ammonia-lyase; 4CL, 4‐coumarate‐CoA ligase; CHS, chalcone synthase; CHI, Chalcone isomerase; F3H, flavonone-3-hydroxylase; F3’H, flavonoid‐3’‐hydroxylase; F3’5’H, flavonoid-3’-5’‐hydroxylase; FLS, flavonol synthase; DFR, dihydroflavonol reductase; ANS, anthocyanidin synthase; LAR, leucocyanidin reductase; ANR, anthocyanidin reductase; UFGT, anthocyanidin 3*-O*-glucosyltransferase; PA, proanthocyanidins. Red and blue colors represent high expression levels of genes.

Supplementary Figure 4 Quantitative real time PCR (qRT-PCR) analysis of some differentially expressed flavonoids biosynthetic structural genes and transcription factor genes. C1H46_001201: PAL (phenylalanine ammonia-lyase); C1H46_013998: 4CL (4‐coumarate‐CoA ligase); C1H46_010805: CHS (chalcone synthase); C1H46_009496: CHI (Chalcone isomerase); C1H46_033494: F3H (flavonone-3-hydroxylase); C1H46_012818: F3’H (flavonoid-3’-5’‐hydroxylase); C1H46_020507: DFR (dihydroflavonol reductase); C1H46_005520: FLS (flavonol synthase); C1H46_043502: UFGT (anthocyanidin 3-O-glucosyltransferase); C1H46_023367 and C1H46_018191: MYB genes; C1H46_000819, C1H46_010137 and C1H46_028789: bZIP genes. The bar plots represent the relative expression levels of genes according to qRT-PCR analysis. The triangles with dashed lines represent the average FPKM values of the genes.

Supplementary Figure 5 Insert size distribution, euclidean distance and venn diagrams of the ATAC-Seq data for fruits at three different ripening stages. For each stage of fruits, two biological replicates were performed.

Supplementary Figure 6 Heatmap of the *MBW* genes identified in differential accessible regions (DARs) showing opposite change pattern in the comparisons of PS5_vs_PS1 and PS9_vs_PS5. A: The *MBW* genes identified in DARs up-regulated in PS5_vs_PS1 and down-regulated in PS9_vs_PS5. B: The *MBW* genes identified in DARs down-regulated in PS5_vs_PS1 and up-regulated in PS9_vs_PS5, the two *bHLH* genes in red were identified in the common DARs.
