# Supplementary figures and images for: Integrated metabolic, transcriptomic and chromatin accessibility analyses provide novel insights into the competition for anthocyanins and flavonols biosynthesis during fruit ripening in red apple
Source: Front Plant Sci. 2022 Sep 23;13:975356. doi: 10.3389/fpls.2022.975356 (PMC9540549; doi:10.3389/fpls.2022.975356)

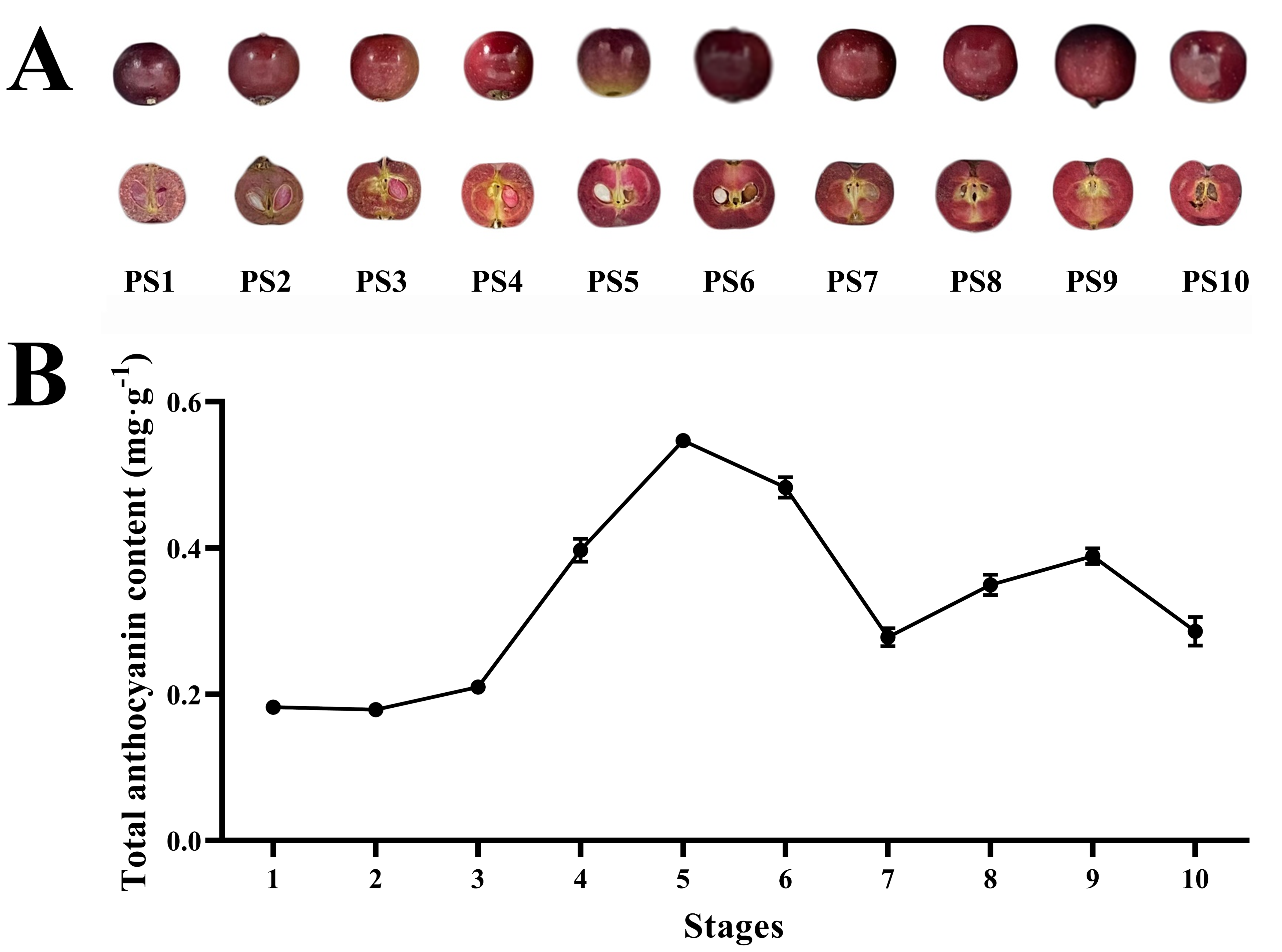

Supplement: Supplementary Figure 1 — Changes in phenotype (A) and anthocyanin accumulation (B) of red apple during fruit ripening. PS1 to P10 represent fruits collected at 7, 9, 11, 13, 15, 17, 19, 21, 23 and 25 weeks post flowering, respectively. There were two anthocyanins accumulation peaks in red apple fruits during ripening, i.e., at PS5 (15 weeks post flowering) and PS9 (23 weeks post flowering), respectively. [file Image_1.png]

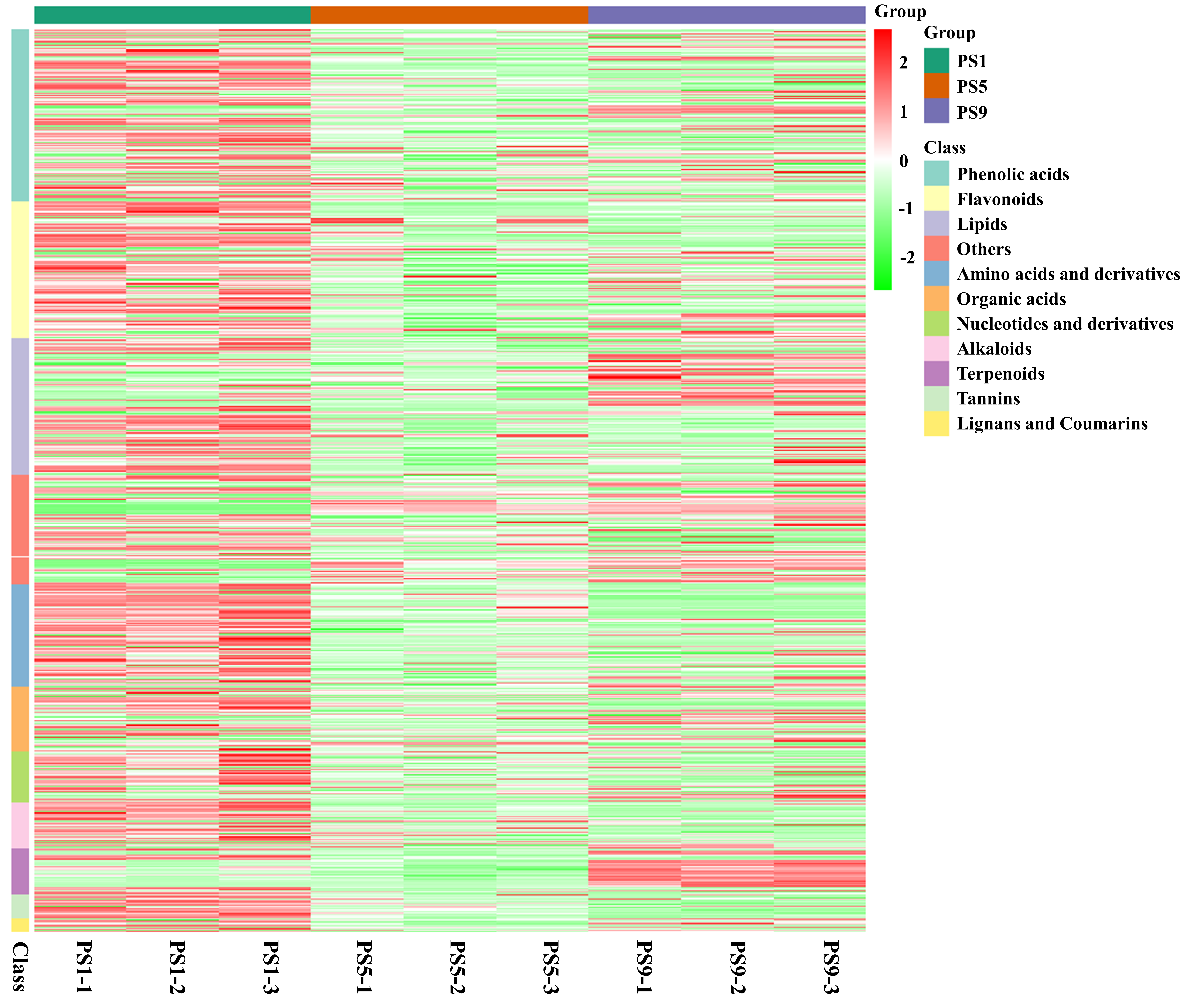

Supplement: Supplementary Figure 2 — Heatmap of clustering of metabolites in red apple fruits at three ripening stages. PS1, PS5 and P9 represent fruits collected at 7, 15 and 23 weeks post flowering, respectively. For each stage, three replicates were performed. Red and green colors represent high and low accumulations, respectively. [file Image_2.png]

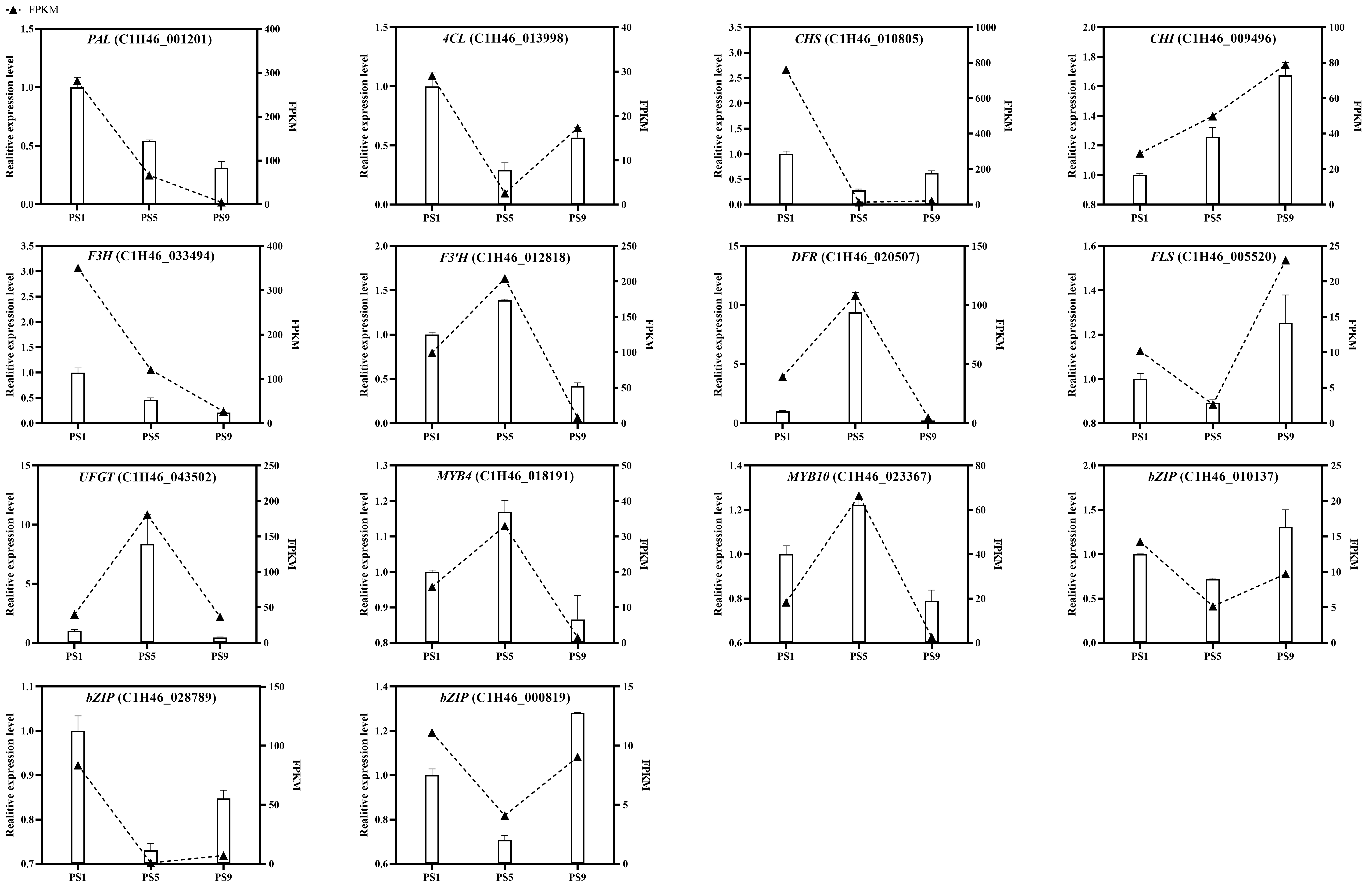

Supplement: Supplementary Figure 4 — Quantitative real time PCR (qRT-PCR) analysis of some differentially expressed flavonoids biosynthetic structural genes and transcription factor genes. C1H46_001201: PAL (phenylalanine ammonia-lyase); C1H46_013998: 4CL (4‐coumarate‐CoA ligase); C1H46_010805: CHS (chalcone synthase); C1H46_009496: CHI (Chalcone isomerase); C1H46_033494: F3H (flavonone-3-hydroxylase); C1H46_012818: F3’H (flavonoid-3’-5’‐hydroxylase); C1H46_020507: DFR (dihydroflavonol reductase); C1H46_005520: FLS (flavonol synthase); C1H46_043502: UFGT (anthocyanidin 3-O-glucosyltransferase); C1H46_023367 and C1H46_018191: MYB genes; C1H46_000819, C1H46_010137 and C1H46_028789: bZIP genes. The bar plots represent the relative expression levels of genes according to qRT-PCR analysis. The triangles with dashed lines represent the average FPKM values of the genes. [file Image_4.png]
